# The systemic immune-inflammation index is superior to predicting clinical remission and relapse for ulcerative colitis patients treated with vedolizumab

**DOI:** 10.3389/fmed.2025.1524307

**Published:** 2025-03-13

**Authors:** Jing Yan, Jun Wu, Rongkun Wang, Pin Meng, Ailing Liu, Yonghong Xu

**Affiliations:** ^1^Department of Gastroenterology, Peking University People's Hospital Qingdao Hospital, Qingdao Women's and Children's Hospital, Qingdao, Shandong, China; ^2^Department of Gastroenterology, The Affiliated Hospital of Qingdao University, Qingdao, Shandong, China; ^3^Department of Vascular Surgery, The Affiliated Hospital of Qingdao University, Qingdao, Shandong, China

**Keywords:** ulcerative colitis, vedolizumab, biomarkers, systemic immuneinflammation index, prognosis

## Abstract

**Background:**

Vedolizumab (VDZ), a novel biologic targeting α4β7 integrin, is safe and effective for the treatment of patients with ulcerative colitis (UC). The objective of this study was to compare the potential of the Platelet-to-lymphocyte ratio (PLR), neutrophil-to-lymphocyte ratio (NLR), and systemic immune-inflammation index (SII) in predicting clinical remission and treatment failure in patients with moderate-to-severe UC on VDZ therapy and to explore the risk factors for treatment failure.

**Methods:**

Seventy-four UC patients treated with VDZ at our institution between December 1, 2020, and October 1, 2023, who had medical records were included in this study. We retrospectively collected baseline NLR, PLR, and SII values and assessed the predictive ability of the three indices for clinical remission and treatment failure using the receiver operating characteristic (ROC) curves.

**Results:**

Patients in the severe group (*n* = 47) had significantly higher baseline PLR and SII values than those in the moderate group (*n* = 27) (*p* < 0.05). Patients with MES3 had significantly higher PLR and SII values than patients with MES2 (*p* < 0.05). At 14 weeks after VDZ treatment, 28 patients obtained steroid-free clinical remission, whereas 46 did not. The area under the ROC curve (AUC) for SII was 0.659 for predicting clinical remission and exhibited the best predictive ability. Of the 52 patients who achieved long-term remission, 35 patients responded consistently to VDZ, whereas 17 patients experienced disease relapse. The SII, with an AUC of 0.793, showed the best predictive ability (sensitivity: 94.1%; specificity: 57.1%; cut-off value: 602.0). Cox regression analysis revealed that SII ≥602.0, was a potential predictor of relapse after VDZ treatment in UC patients (*p* = 0.048, hazard ratio: 8.651; 95% confidence interval: 1.017–73.593).

**Conclusion:**

The SII performed better than NLR and PLR in predicting clinical remission and relapse for UC patients on VDZ therapy. Moreover, patients with high SII may relapse after VDZ treatment and should be treated with caution.

## Introduction

Ulcerative colitis (UC) is an autoimmune chronic bowel disease that affects the quality of life and longevity of patients, and the prevalence of the disease in Asian populations has been on the rise in recent years (1, 2). Conventional medications such as 5-aminosalicylates show limited utility in the management of moderate-to-severe UC (1). Although corticosteroids can be used for induction therapy in moderate-to-severe UC, some patients with steroid-refractory or steroid-dependent colitis have poor efficacy (3, 4). Immunomodulators such as azathioprine have been shown to have serious side effects such as myelosuppression and opportunistic infection (5). Anti-tumor necrosis factor (TNF) agents and other biologics are novel medications for inducing and maintaining endoscopic and clinical remission in moderate-to-severe UC, with the promise of improving prognosis and reducing hospitalization and colectomy rates (6, 7).

Vedolizumab (VDZ), a novel biologic targeting α4β7 integrin, has been reported to be safe and effective for UC patients in the induction and maintenance of endoscopic and clinical remission (8). The effectiveness of VDZ has been confirmed in domestic and international reports, but there are still patients who do not respond to VDZ and are forced to turn to other biologic therapies or colectomy (9). Prolonged refractory treatment unnecessarily extends the duration of therapy and increases the financial burden of medical care for families and society. Therefore, in cases of refractory therapy, a decision to switch to another therapy should be made quickly and accurately.

Currently, neutrophil-to-lymphocyte ratio (NLR), platelet-to-lymphocyte ratio (PLR) and systemic immune-inflammation index (SII) obtained from complete blood count are novel indices for assessing systemic inflammatory status and disease activity and can predict treatment outcomes and prognosis in patients with inflammatory diseases like UC (10–12). NLR and PLR were initially reported to be associated with the prognosis of rheumatic diseases, coronary artery disease and malignant tumors (13–17). Recently, Posul et al. found a correlation between NLR and active UC, while Nishida et al. demonstrated that NLR could predict treatment failure of infliximab in active UC (10, 18). Bertani et al. reported that PLR predicted mucosal remission and long-term clinical remission in UC patients receiving anti-TNF therapy, but its efficacy was weaker than NLR (19). The SII, which was proposed by Hu et al. in 2014, was associated with the active status and prognosis of autoimmune diseases, cardiovascular diseases, and malignancies (20–22). Xie et al. discovered that SII was significantly increased in active UC and was strongly associated with disease activity (12). In addition, it showed a favorable association with Mayo endoscopy subscore (MES), clinical Mayo score and C-reactive protein (CRP) (23, 24).

To our knowledge, there are no reports evaluating the predictive value of NLR, PLR and SII for the prognosis of UC patients treated with VDZ. Therefore, the aim of this study was to compare the predictive potential of the NLR, PLR, and SII for clinical remission and treatment failure after VDZ treatment in patients with moderate-to-severe UC and to identify risk factors contributing to treatment failure in patients.

## Materials and methods

### Patients

**Figure 1:**
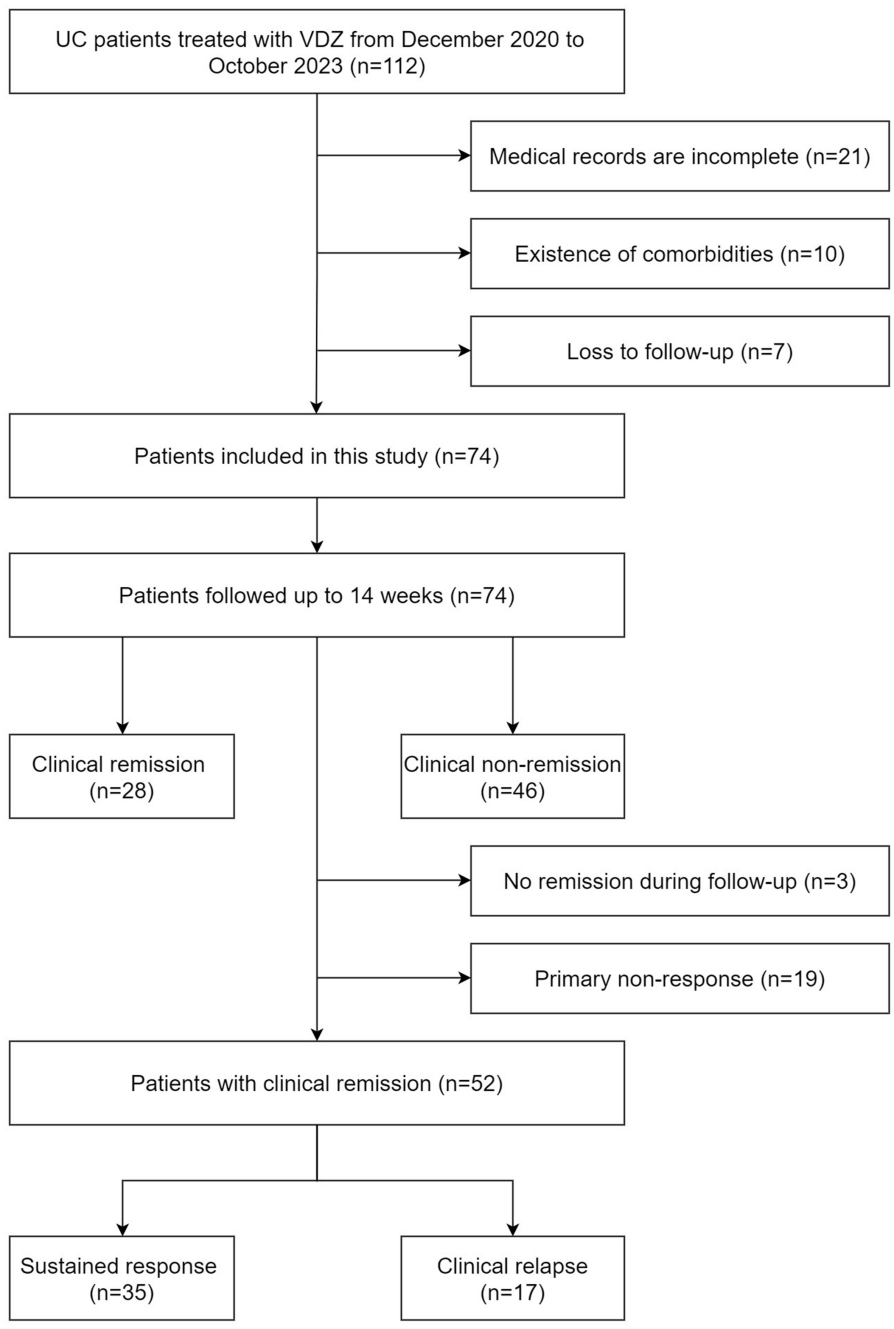
Flow chart for enrollment of the study population (*n* = 74).

We enrolled UC patients who received VDZ at the Affiliated Hospital of Qingdao University (Shandong Province, China) between December 1, 2020, and October 1, 2023 with medical records in our study. The diagnosis of UC was made based on clinical presentation, colonoscopy, and histological evaluation, as well as the exclusion of patients with intestinal tuberculosis, Crohn’s disease, and unclassified inflammatory bowel disease (IBD) (1). Other inclusion criteria were as follows: (a) Moderate-to-severe UC patients according to partial Mayo score (PMS) and/or MES (PMS ≥5 or MES ≥2); (b) colonoscopy performed at our institution before treatment, and laboratory data obtained within 1 week before the first VDZ infusion; and (c) no comorbidities such as active infectious diseases, cardiovascular diseases, malignant tumors, and autoimmune diseases. A detailed flowchart of the patient selection process is shown in Figure 1. Ultimately, 74 patients were included in this retrospective study. According to the recommended dosage regimen for VDZ, the induction treatment was administered at 300 mg at weeks 0, 2, and 6, followed by maintenance treatment for 8 weeks.

### Data collection

The baseline and follow-up data of enrolled patients were obtained by reviewing the hospital medical record system and by telephone consultation, including the following: (a) gender, age at diagnosis, duration of disease, history of smoking, and use of drugs (both previous treatment and concomitant medication); (b) colonoscopy data, such as the extent of lesions and MES; (c) the daily number of stools, blood in feces, and overall physician assessment were collected at regular follow-up visits to obtain the PMS; and (d) laboratory markers such as CRP, hemoglobin, albumin, platelet count, neutrophil count, and lymphocyte count.

This retrospective study was conducted following the principles of clinical practice of the Declaration of Helsinki and was approved by the Ethics Committee of the Affiliated Hospital of Qingdao University (QYFY WZLL 28425). This study got exemption from informed consent because of the retrospective study design.

### Clinical and endoscopic assessment

The Truelove and Witts severity index was used to determine the severity of patients’ disease, categorizing them as mild, moderate, and severe (25). PMS (range: 0–12) was collected at regular follow-up visits prior to each infusion to assess response to VDZ in UC patients (26). Definition of clinical response was a decrease of at least 3 points in PMS (along with a decrease of ≥30% from baseline) and a blood in stool score of ≤1 or a decrease of 1 point. Clinical remission was considered as a PMS ≤2, with no sub-score above 1 (27). Steroid-free clinical remission was defined as clinical remission with no combination of steroids. Relapse was defined as a change in disease from a period of clinical remission to an active phase, which was identified as a PMS of >2 (28).

Colonoscopy was performed to assess the mucosal status of all included patients before VDZ induction. MES was evaluated according to the following rules: 0 was classified as normal or inactive mucosa; 1 was classified as a mild lesion showing reduced vascular pattern, erythema, and mild friability; 2 was classified as a moderate lesion showing lack of vascular pattern, marked erythema, friability, and erosions; and 3 was classified as a severe lesion showing spontaneous hemorrhage and ulceration (29).

### Laboratory indicators

Baseline laboratory data, including complete blood counts, were obtained within 1 week before the first VDZ infusion. NLR is equal to the neutrophil count divided by the lymphocyte count, PLR is the ratio of the platelet count to the lymphocyte count, and SII is calculated as follows: SII = (platelet count×neutrophil count)/lymphocyte count.

### Outcomes

The primary endpoint was clinical relapse by assessing and comparing the early predictive value of NLR, PLR, and SII for relapse in UC patients receiving VDZ therapy. The secondary endpoint was to assess the predictive ability of the three biomarkers for clinical remission.

### Statistical analysis

Continuous and categorical variables were presented as median (interquartile range [IQR]) and number (frequency), respectively. The Mann–Whitney U test was employed to compare nonparametric variables, while the chi-square test or Fisher’s exact test categorical data was examined for comparisons of categorical data. Spearman’s rank correlation coefficient was applied to compare the correlations between biomarkers and MES, PMS, and CRP levels. The predictive value of NLR, PLR, and SII for clinical remission and treatment failure were assessed and compared using receiver operating characteristic (ROC) curve analysis. Finally, the hazard ratio (HR) and 95% confidence interval (CI) of SII for VDZ treatment failure in UC patients were determined using Cox proportional risk regression, and the Kaplan–Meier method was used to calculate the cumulative clinical relapse-free survival rate. Statistical analyses were performed using SPSS (version 26.0, SPSS Inc.) software, and GraphPad Prism 5 software was applied for graphing. Differences were considered statistically significant at *p* < 0.05.

## Results

### Patient characteristics

The baseline characteristics of 74 patients included in this study are shown in Table 1. The number of male patients was 50, with a percentage of 67.6%. The median age at diagnosis and disease duration of the patients was 44.0 years (range: 10.0–73.0 years) and 4.50 years (range: 0.08–30.00 years), respectively. Thirteen patients had a history of smoking. Based on the Montreal classification, E1, E2, and E3 had 1, 12, and 61 cases, respectively. Twenty-seven patients had moderate colitis, and 47 patients had severe colitis. Fourteen patients had an MES of 2 and 60 patients had an MES of 3. Twenty-seven patients (36.5%) were combined corticosteroids. 24 (32.4%), 3 (4.1%), and 6 (8.1%) patients had been received with corticosteroids, immunosuppressants, and anti-TNF agents, respectively. The baseline laboratory data, such as the NLR, PLR, and SII, are presented in Table 1.

**Table 1:** Clinical characteristics of ulcerative colitis patients at baseline.

Characteristics	Total (*N* = 74)
Male gender, *n* (%)	50 (67.6)
Age at diagnosis, years, median (range)	44.0 (10.0–73.0)
Disease duration, years, median (range)	4.50 (0.08–30.00)
History of smoking, *n* (%)	13 (17.6)
Extent of disease, *n* (%)	
E1	1 (1.4)
E2	12 (16.2)
E3	61 (82.4)
Truelove and Witts severity index, *n* (%)	
Moderate	27 (36.5)
Severe	47 (63.5)
MES, *n* (%)	
MES 2	14 (18.9)
MES 3	60 (81.1)
Previous medications, *n* (%)	
Corticosteroids	24 (32.4)
Anti-TNF agents	6 (8.1)
Immunosuppressants	3 (4.1)
Concomitant medications, *n* (%)	
Corticosteroids	27 (36.5)
Partial Mayo score	7.5 (6.0–8.0)
CRP, mg/L	6.02 (1.20–14.43)
Hemoglobin, g/L	118.5 (98.0–137.8)
Albumin, g/L	37.48 (32.1–41.3)
Neutrophil count, ×10^9^/L	4.43 (3.55–5.98)
Platelet count, ×10^9^/L	274.5 (234.3–352.0)
NLR	2.34 (1.87–3.49)
PLR	151.3 (111.3–223.6)
SII, ×10^9^/L	645.1 (457.4–1136.0)

Results are expressed as: frequency (%) or median (range) or median (IQR). MES, Mayo endoscopy subscore; TNF, tumor necrosis factor; IQR, interquartile range; CRP, C-reactive protein; NLR, neutrophil-to-lymphocyte ratio; PLR, platelet-to-lymphocyte ratio; SII, systemic immune-inflammation index.

### Correlations between the NLR, PLR, SII and clinical/endoscopic/laboratory parameters

The associations between the NLR, PLR, SII and clinical/endoscopic/laboratory parameters are shown in Table 2. The NLR and PLR were associated with PMS (*r* = 0.236 ~ 0.324, *p* < 0.05). The PLR and SII showed positive correlations with CRP and MES (*r* = 0.236 ~ 0.368, *p* < 0.05), and negative correlations with hemoglobin and albumin (*r* = −0.364 ~ −0.319, *p* < 0.01). Additionally, NLR was weakly associated with CRP (*r* = 0.404, *p* < 0.001) and albumin (*r* = −0.266, *p* = 0.022), but not associated with hemoglobin or MES (*p* > 0.05).

**Table 2:** Correlations between NLR, PLR, SII and clinical/endoscopic/laboratory parameters in 74 patients with ulcerative colitis.

Parameters	NLR		PLR		SII	
	*r*	*p* value	*r*	*p* value	*r*	*p* value
Partial mayo score	0.236	0.043	0.324	0.005	0.182	0.121
CRP	0.404	<0.001	0.366	0.002	0.368	0.002
Hemoglobin	−0.129	0.274	−0.364	0.001	−0.338	0.003
Albumin	−0.266	0.022	−0.319	0.006	−0.324	0.005
MES	0.116	0.324	0.237	0.042	0.236	0.043

CRP, C-reactive protein; NLR, neutrophil-to-lymphocyte ratio; PLR, platelet-to-lymphocyte ratio; SII, systemic immune-inflammation index; MES, Mayo endoscopy subscore.

### Comparison of the NLR, PLR and SII in different disease severities of UC patients receiving VDZ

**Figure 2:**
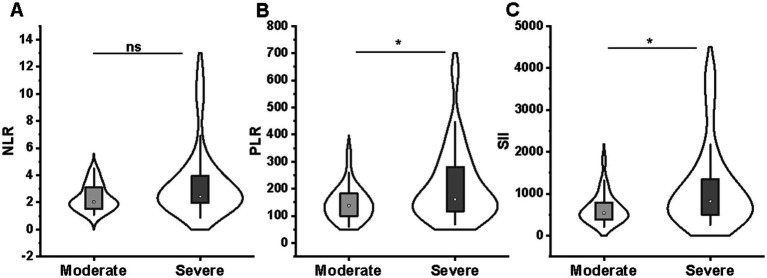
Comparison of NLR **(A)**, PLR **(B)**, and SII **(C)** between moderate (*n* = 27) and severe (*n* = 47) groups. NLR **(A)** values showed no difference between the moderate and severe groups; patients in the severe group had a significantly higher PLR **(B)** and SII **(C)** at baseline than patients with moderate colitis. NLR, neutrophil-to-lymphocyte ratio; PLR, platelet-to-lymphocyte ratio; SII, systemic immune-inflammation index.

We categorized patients into the moderate (*n* = 27) and severe (*n* = 47) groups based on the Truelove-Witts Severity Index. It was found that patients in the severe group had a significantly higher PLR (median [IQR]:161.2 [117.2–280.4] vs. 137.7 [99.6–183.3]; *p* = 0.044) and SII (821.9 [498.5–1343.8] vs. 540.5 [384.1–787.1]; *p* = 0.029) at baseline than patients with moderate colitis (Figures 2B,C). But NLR values showed no difference between the moderate and severe groups (2.44 [1.95–3.94] vs. 2.04 [1.52–3.10]; *p* = 0.051) (Figure 2A).

**Figure 3:**
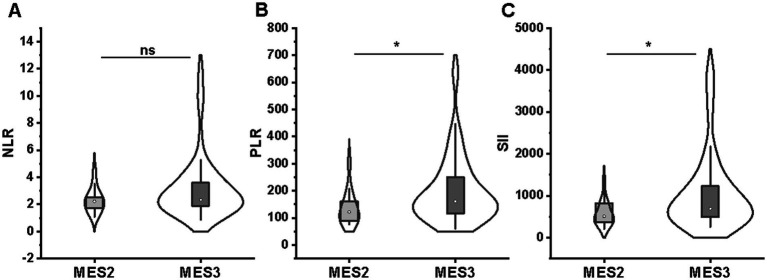
Comparison of NLR **(A)**, PLR **(B)**, and SII **(C)** between MES 2 (*n* = 14) and MES 3 (*n* = 60) groups. No difference in NLR **(A)** values between MES subgroups; patients with MES 3 had significantly higher PLR **(B)** and SII **(C)** values than patients with MES 2. NLR, neutrophil-to-lymphocyte ratio; PLR, platelet-to-lymphocyte ratio; SII, systemic immune-inflammation index; MES, Mayo endoscopy subscore.

For endoscopic evaluation at baseline, patients with deep ulcers (MES = 3) had significantly higher PLR values (160.8 [115.7–255.1] vs. 122.1 [87.0–166.0]; *p* = 0.043) and SII values (693.5 [492.4–1241.0] vs. 506.1 [364.9–828.1]; *p* = 0.045) than patients with shallow ulcers (MES = 2) (Figures 3B,C). However, no difference in NLR values between MES subgroups (2.34 [1.89–3.61] vs. 2.22 [1.67–2.64]; *p* = 0.324) (Figure 3A).

### Comparison of the NLR, PLR, and SII in predicting 14-week steroid-free clinical remission for UC patients receiving VDZ

**Figure 4:**
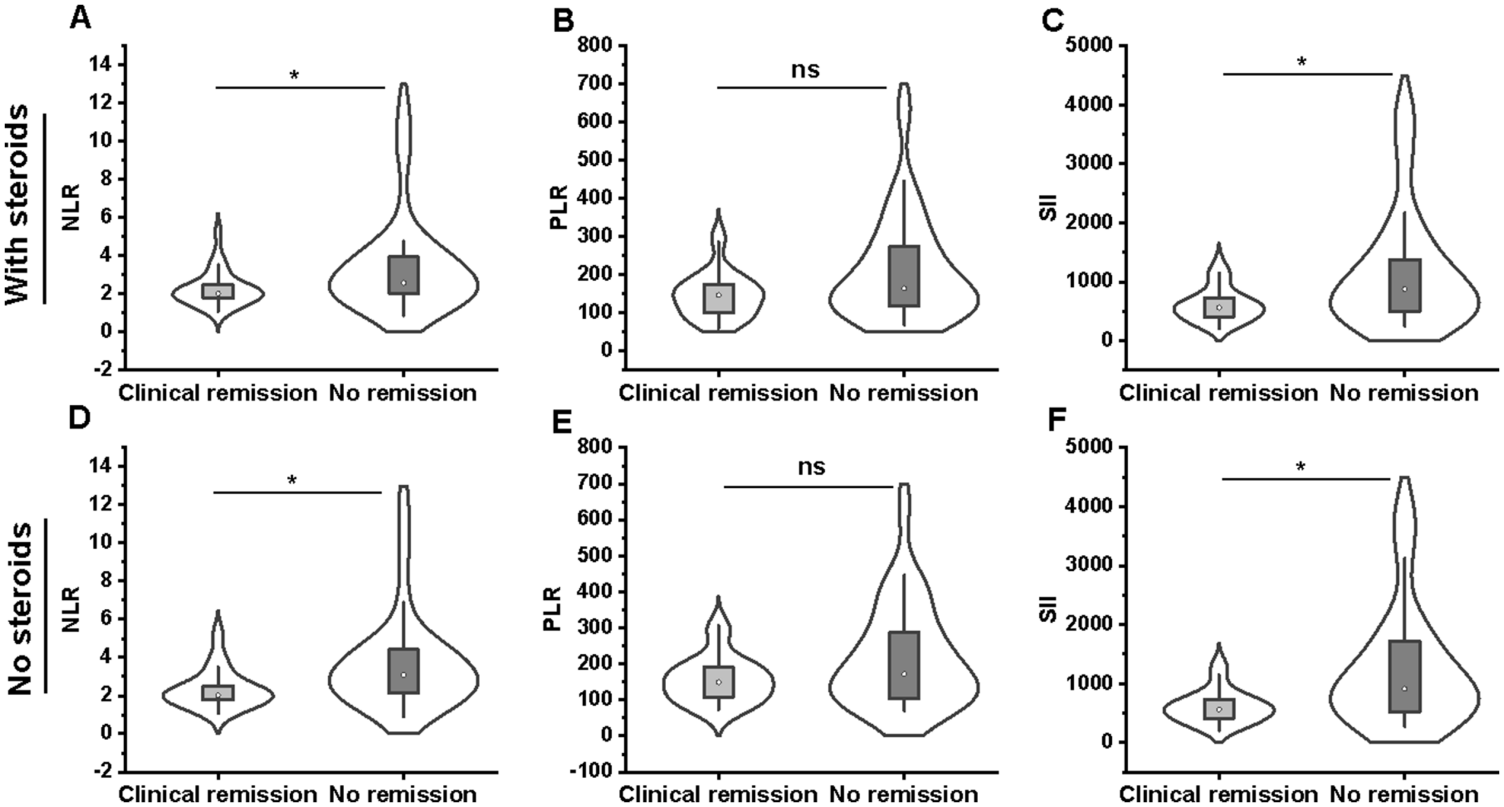
Comparison of NLR **(A)**, PLR **(B)**, and SII **(C)** between steroid-free clinical remission (*n* = 28) and non-remission (*n* = 46) groups. Comparison of NLR **(D)**, PLR **(E)**, and SII **(F)** between clinical remission (*n* = 20) and non-remission (*n* = 27) groups for patients undergoing VDZ therapy without a combination of steroids. NLR, neutrophil-to-lymphocyte ratio; PLR, platelet-to-lymphocyte ratio; SII, systemic immune-inflammation index.

Of the 74 included patients, 28 patients achieved 14-week steroid-free clinical remission, and 46 patients were not in remission after VDZ treatment. We used the Mann–Whitney U test to compare NLR, PLR, and SII values between clinical remission and non-remission groups. Patients who obtained clinical remission had lower levels of NLR (2.02 [1.75–2.48] vs. 2.57 [2.00–3.97]; *p* = 0.026) and SII (562.2 [395.4–750.2] vs. 877.1 [492.1–1400.9]; *p* = 0.023) than those who did not experience remission, while there was no significant difference in PLR values between remission and non-remission groups (146.9 [99.7–179.6] vs. 164.4 [117.0–275.4]; *p* = 0.071) (Figures 4A–C). In addition, similar results were observed in patients undergoing VDZ therapy without a combination of steroids (Figures 4D–F).

**Figure 5:**
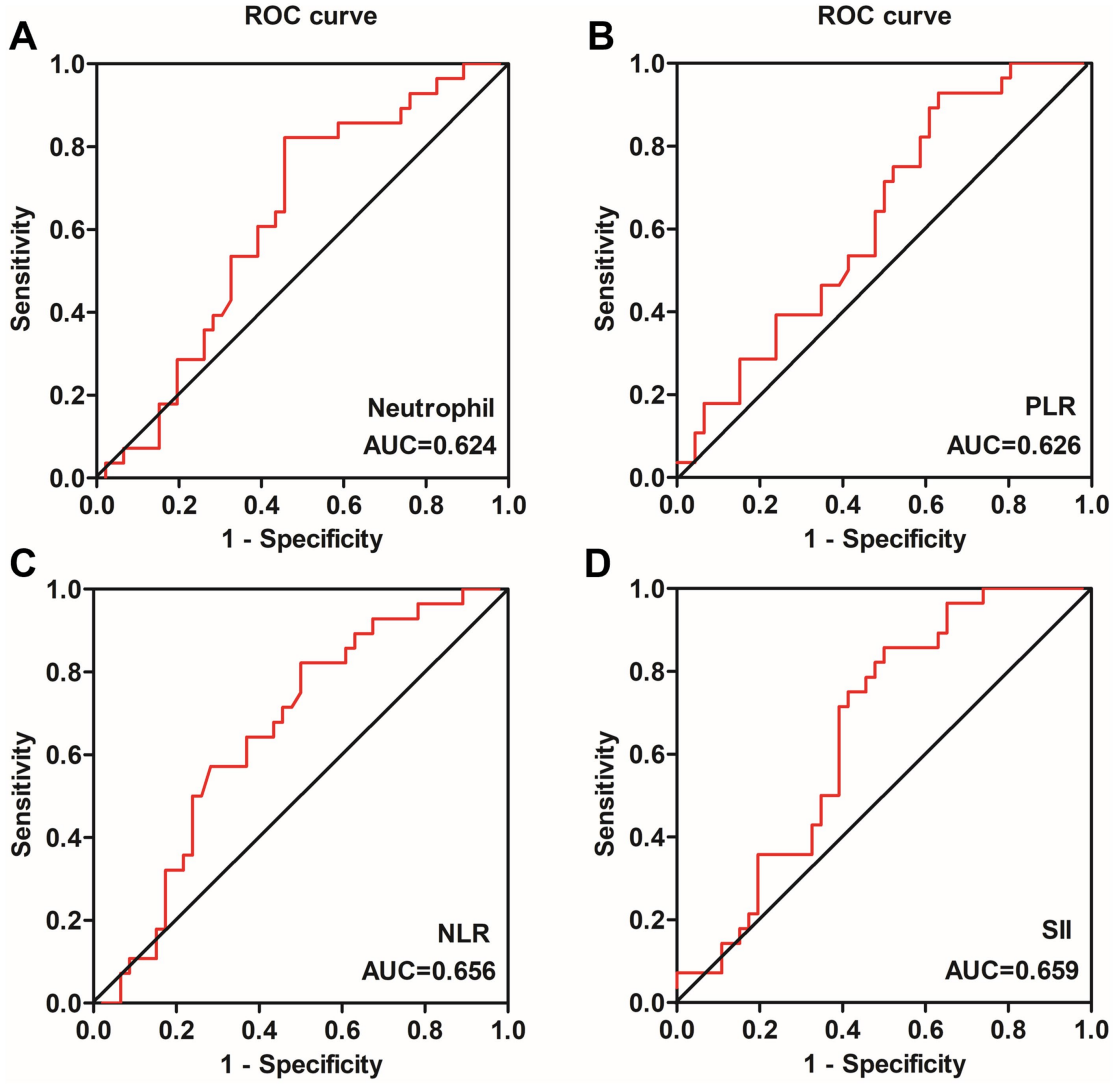
ROC curves of the neutrophil count **(A)**, PLR **(B)**, NLR **(C)** and SII **(D)** in predicting 14-week steroid-free clinical remission in ulcerative colitis patients. The SII **(D)** exhibited the best predictive ability for predicting 14-week clinical remission, the NLR **(C)** had a lower predictive ability, while the neutrophil count **(A)** and PLR **(B)** had no potential. NLR, neutrophil-to-lymphocyte ratio; PLR, platelet-to-lymphocyte ratio; SII, systemic immune-inflammation index; ROC, receiver operating characteristic.

We subsequently performed ROC analysis to comparatively assess the predictive potential of the neutrophil count, NLR, PLR, and SII for 14-week clinical remission. The area under the ROC curve (AUC) for SII (sensitivity = 85.7%, specificity = 50.0%, cut-off value = 880.0) was 0.659 for predicting 14-week clinical remission, exhibiting the best predictive ability (*p* = 0.022) (Figure 5D). The NLR, with an AUC of 0.656, had a lower predictive ability (sensitivity = 82.1%, specificity = 50.0%, cut-off value = 2.62) (*p* = 0.025) (Figure 5C). However, the neutrophil count and PLR had no potential to predict 14-week clinical remission (*p* > 0.05) (Figures 5A,B).

However, 27 of the 74 patients were treated with VDZ combined with systemic corticosteroids, which may have affected the NLR, PLR, and SII. In further analysis, no association was found between the three biomarkers and concomitant steroid therapy (Supplementary Figures 1A–C).

### Comparison of the NLR, PLR, and SII in predicting relapse for UC patients receiving VDZ

**Figure 6:**
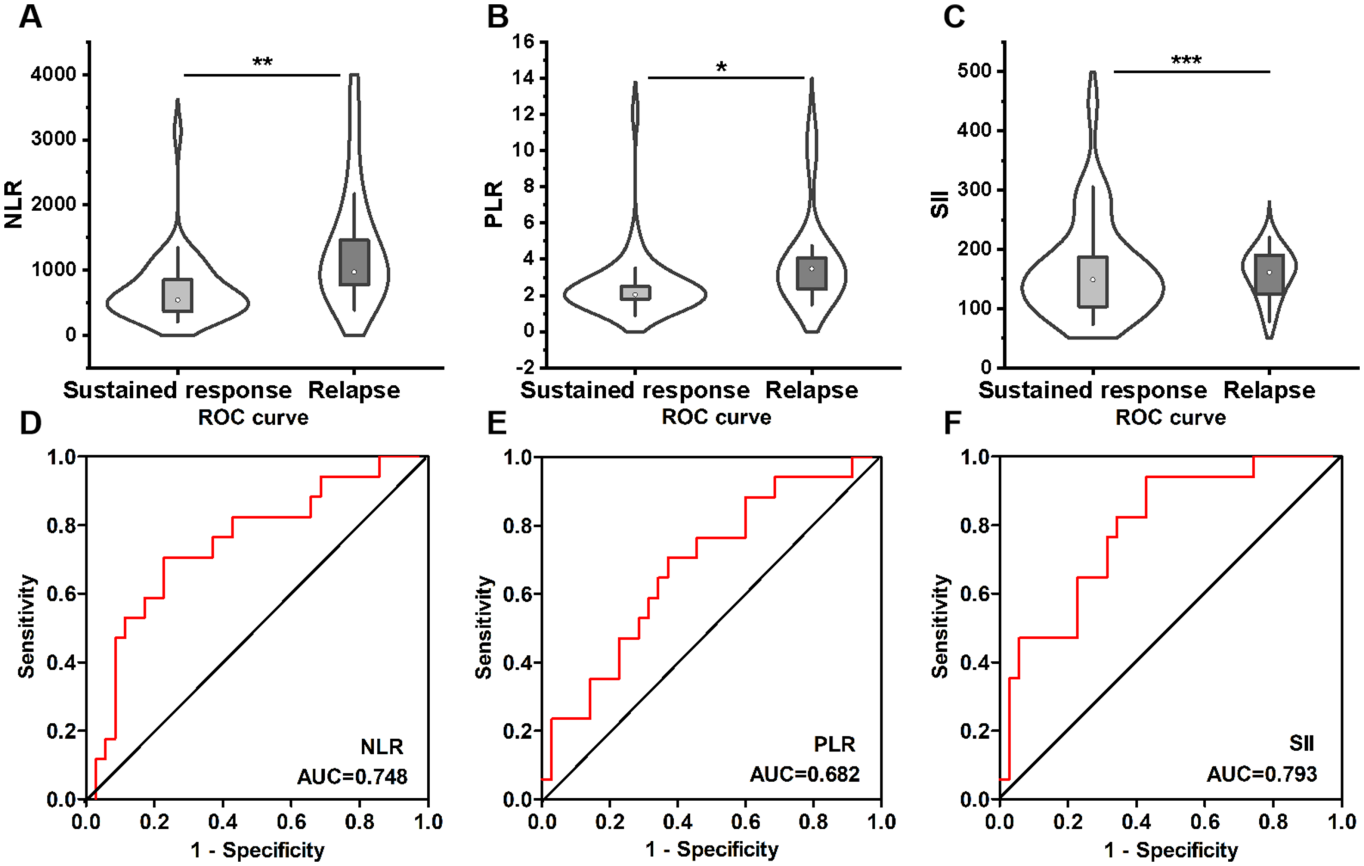
Comparison of NLR **(A)**, PLR **(B)**, and SII **(C)** between sustained response (*n* = 35) and relapse (*n* = 17) groups. ROC curves of NLR **(D)**, PLR **(E)** and SII **(F)** in predicting relapse in ulcerative colitis patients. NLR, neutrophil-to-lymphocyte ratio; PLR, platelet-to-lymphocyte ratio; SII, systemic immune-inflammation index; ROC, receiver operating characteristic.

Of the 52 patients who achieved long-term remission, 35 patients had a sustained response to VDZ, whereas 17 patients experienced disease relapse during the follow-up period. The patients who experienced disease relapse had significantly higher baseline NLR (3.48 [2.28–4.08] vs. 2.04 [1.80–2.49]; *p* = 0.004), PLR (183.0 [141.1–290.8] vs. 148.4 [99.6–187.4]; *p* = 0.034) and SII (971.8 [719.8–1738.8] vs. 540.5 [366.7–852.5]; *p* = 0.001) values than those with sustained response to VDZ (Figures 6A–C).

Subsequently, the optimal cut-off values for NLR, PLR, and SII were determined to predict disease relapse using the ROC curve. The best capacity for predicting disease relapse was achieved with an SII value of 602.0 (AUC = 0.793, sensitivity = 94.1%, specificity = 57.1%) (*p* = 0.001) (Figure 6F). Moreover, the NLR showed a good predictive ability, with an AUC of 0.748 (sensitivity = 70.6%, specificity = 77.1%, cut-off value = 2.52) (*p* = 0.004) (Figure 6D). The indicator with a lower predictive potential was the PLR with an AUC of 0.682 (sensitivity = 70.6%, specificity = 62.9%, cut-off value = 160.5) (*p* = 0.034) (Figure 6E). This indicates that baseline SII may offer more advantages in predicting treatment failure in UC patients receiving VDZ therapy.

### The SII is an independent factor for predicting relapse in UC patients receiving VDZ

In univariate analysis, clinical activity (HR: 3.197; 95% CI: 1.037–9.858; *p* = 0.043), response at 14 weeks (HR: 0.274; 95% CI: 0.089–0.843; *p* = 0.024), and SII (HR: 13.109; 95% CI: 1.737–98.900; *p* = 0.013) were associated with relapse after VDZ treatment in UC patients. While Cox regression analysis showed that SII ≥602.0 was an independent risk factor for predicting relapse (adjusted HR: 8.651; 95% CI: 1.017–73.593; *p* = 0.048). Other factors such as gender, disease duration, age at diagnosis, smoking history, extension of disease, MES, and combined corticosteroid use, were not associated with long-term prognosis (Table 3).

**Table 3:** Cox regression analysis for predicting clinical relapse during follow-up after vedolizumab induction therapy.

Variables	Relapse, n/N (%)	Unadjusted HR (95% CI)	*p* value	Adjusted HR (95% CI)	*p* value
Gender		0.851 (0.275–2.627)	0.778		
Male	13/37 (35.1)				
Female	4/15 (26.7)				
Age at diagnosis, years		1.431 (0.464–4.411)	0.533		
≤ 50	13/36 (36.1)				
> 50	4/16 (25.0)				
Disease duration, years		0.711 (0.267–1.893)	0.495		
≤ 5	9/28 (32.1)				
> 5	8/24 (33.3)				
History of smoking		1.339 (0.382–4.689)	0.648		
Yes	3/9 (33.3)				
No	14/43 (32.6)				
Extent of disease		1.230 (0.377–4.010)	0.731		
E1	0/1 (0)				
E2	3/10 (30.0)				
E3	14/41 (34.1)				
Clinical activity		3.197 (1.037–9.858)	**0.043**	1.347 (0.412–4.403)	0.622
Moderate	4/23 (17.4)				
Severe	13/29 (44.8)				
MES		5.365 (0.711–40.501)	0.103		
MES 2	1/12 (8.3)				
MES 3	16/40 (40.0)				
Concomitant steroids		2.141 (0.824–5.563)	0.118		
Yes	9/18 (50.0)				
No	8/34 (23.5)				
Response at 14 weeks		0.274 (0.089–0.843)	**0.024**	0.497 (0.156–1.584)	0.237
Clinical remission	4/28 (14.3)				
Non-remission	13/24 (54.2)				
SII, ×10^9^/L		13.109 (1.737–98.900)	**0.013**	8.651 (1.017–73.593)	**0.048**
≥ 602.0	16/31 (51.6)				
< 602.0	1/21 (4.8)				

Bold values are expressed as: *p* value <0.05. CI, confidence interval; HR, hazard ratio; MES, Mayo endoscopy subscore; SII, systemic immune-inflammation index.

**Figure 7:**
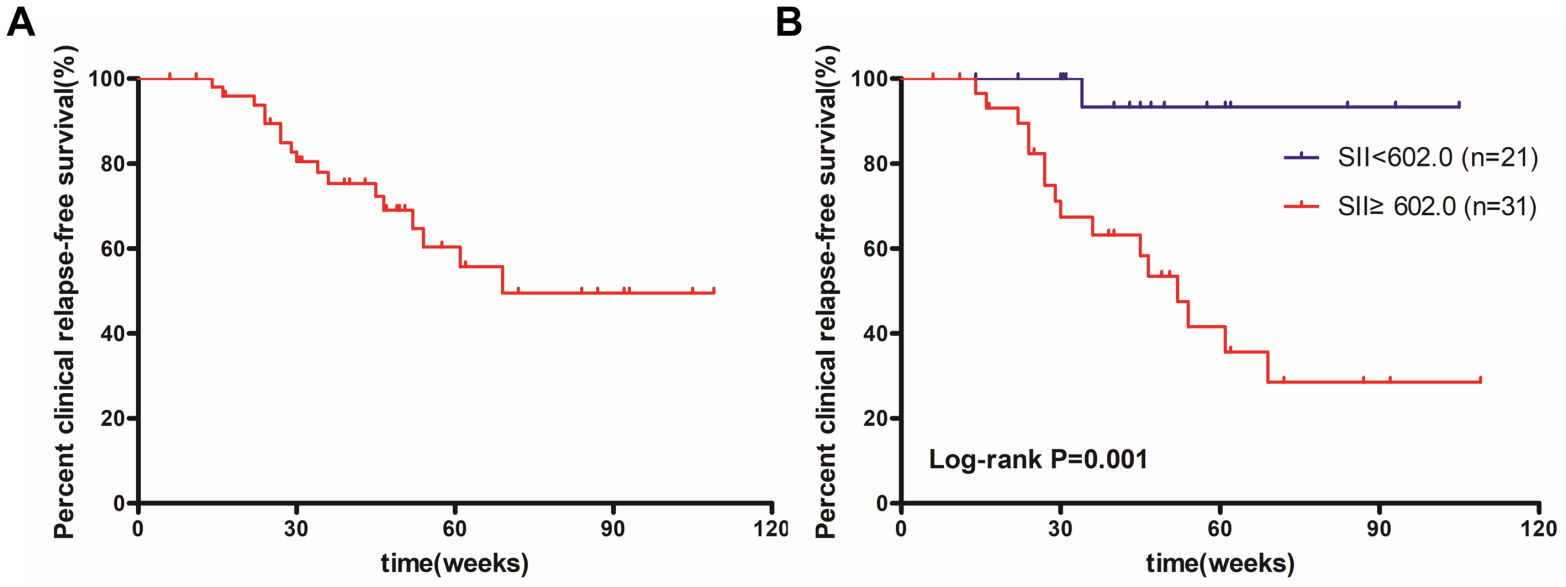
Kaplan–Meier curves of clinical relapse-free survival for 52 patients **(A)** and patients with SII <602.0 and SII ≥602.0 **(B)**. SII, systemic immune-inflammation index.

According to the ROC curve analysis of the SII, we categorized 52 patients into low SII group (*n* = 21) and high SII group (*n* = 31). Table 4 shows the demographic and biochemical data for each category. The differences in PMS, CRP, hemoglobin, and albumin values between patients with high and low SII were statistically significant. Cumulative relapse-free survival after VDZ treatment is shown in Figure 7. The range of follow-up period was 11–122 weeks. Cumulative relapse-free survival rates were 89.4% at 6 months and 60.4% at 12 months. Importantly, patients in the high SII group had a significantly lower rate of relapse-free survival than those in the low SII group (*p* = 0.001) (Figures 7A,B). 51.6% of patients with high SII values experienced disease recurrence during follow-up, compared with only one patient in the low SII group.

**Table 4:** Patient demographics and biochemical data based on SII ≥602.0 and SII <602.0.

SII, ×10^9^/L	≥602.0 (*n* = 31)	<602.0 (*n* = 21)	*p* value
Gender: male/female	22/9	15/6	0.971
Age at diagnosis, years	44.0 (31.0–53.0)	40.0 (25.0–54.0)	0.544
Disease duration, years	5.0 (3.0–8.0)	3.0 (1.0–10.0)	0.519
Partial Mayo score	8.0 (6.0–8.0)	6.0 (5.0–8.0)	0.039
CRP, mg/L	7.18 (2.04–18.36)	1.14 (0.65–5.08)	0.002
Hemoglobin, g/L	120.0 (97.0–136.0)	134.0 (119.0–153.0)	0.017
Albumin, g/L	35.55 (32.07–38.32)	41.13 (39.06–44.21)	<0.001

Results are expressed as: median (IQR). SII, systemic immune-inflammation index; CRP, C-reactive protein.

**Figure 8:**
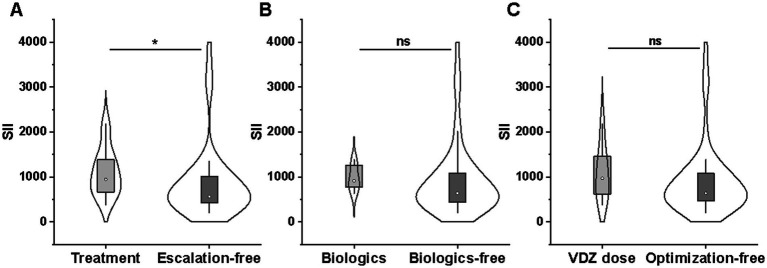
Comparison of SII between treatment escalation (*n* = 14) and escalation-free (*n* = 38) groups **(A)**, biologics (*n* = 6) and biologics-free (*n* = 46) groups **(B)**, and VDZ-dose optimization (*n* = 7) and optimization-free (*n* = 45) groups **(C)**. SII, systemic immune-inflammation index; VDZ, Vedolizumab.

Fourteen of the 17 relapsed patients underwent treatment escalation, including VDZ dose optimization in seven cases, anti-TNF agents in four cases (two infliximab and two mirikizumab), tofacitinib in two cases, and steroids in one case. Overall, the SII was significantly higher in patients receiving treatment escalation (949.7 [658.7–1400.9] vs. 562.2 [413.6–1031.7]; *p* = 0.012), but no correlation was found between the SII and VDZ-dose optimization/biologics (*p* > 0.05) (Figures 8A–C).

## Discussion

In this study, the clinical utility of the baseline NLR, PLR, and SII in UC patients treated with VDZ was explored and compared. Notably, although NLR and SII exhibited similar potential for predicting short-term clinical remission, NLR did not correlate with clinical activity and MES in response to the degree of mucosal inflammation. Furthermore, it was demonstrated that the SII is a potential biomarker for predicting relapse in patients with moderate-to-severe UC after VDZ-induced therapy. Patients with a baseline SII > 602.0, had a higher risk of relapse after clinical remission on VDZ therapy compared to patients with a low SII. Thus, SII is superior to NLR and PLR in predicting treatment outcomes and reflecting disease activity in UC treated with VDZ and can be used to guide clinical decision-making.

VDZ is a monoclonal antibody specifically targeting α4β7 integrins located on the surface of circulating lymphocytes and can be used in IBD patients who had failed or intolerant to anti-TNF (30). However, approximately 18–39% of UC patients receiving VDZ experienced loss of response and required treatment escalation, such as switching to another biologic or colectomy (31). Therefore, early prediction of therapeutic response and prognosis of patients with moderate-to-severe UC to VDZ therapy is critical in helping clinicians make decisions about treatment choices. The associations between NLR, PLR, SII and active UC have been demonstrated in several studies (24, 32). Bertani and Nishida et al. showed that NLR and PLR could be potential predictors of treatment outcome with biologics (18, 19). However, no studies have analyzed and compared the value of NLR, PLR, and SII in predicting treatment outcomes in UC patients receiving VDZ therapy.

Neutrophils are one of the key inflammatory cells involved in UC pathogenesis. Neutrophils can cause mucosal ulceration by mass infiltration into areas of intestinal inflammation, releasing protein-hydrolyzing enzymes, oxygen free radicals, and other substances that damage crypt tissue (33). In addition, studies have demonstrated abnormalities in peripheral lymphocyte function in patients with IBD, and the absolute lymphocyte counts are considered to be an indicator of the reactive level of the body’s immune system (34, 35). Platelet count, a potential biomarker, correlates with the degree of endoscopic inflammation and disease recurrence in UC patients, and is significantly higher for patients with active UC than those in the remission stage (36). SII, as a novel biomarker based on neutrophil, platelet, and lymphocyte counts, is readily available and cost-effective (37).

The association between SII and active UC has been explored in previous studies (12, 23, 24). In a retrospective study by Xie et al. (12), it was found that SII levels were positively correlated with MES, CRP, and ESR, with a specificity of 0.750, and a sensitivity of 0.641 for diagnosing active UC when SII was >485.95. Pakoz et al. reported that patients with active UC had significantly higher SII than patients in remission (23). They both concluded that SII is a non-invasive marker of active UC. Similarly, the results of our study also demonstrated that the SII was significantly correlated with MES and laboratory parameters (CRP, hemoglobin, and albumin). Furthermore, significant differences in CRP, hemoglobin, and albumin between the high SII and low SII groups again proved their association. SII levels can reflect the endoscopic severity of UC, and patients with more severe intestinal mucosal inflammation have an increased chance of developing anemia and hypoalbuminemia.

This study demonstrated that NLR, PLR, and SII were able to differentiate between moderate and severe colitis, which is essential for predicting disease severity and developing treatment strategies. In the assessment of endoscopic mucosal inflammation, patients with an MES of 3 had significantly higher SII and PLR values than those with an MES of 2. However, the NLR did not show significant differences between MES subgroups and could not differentiate between shallow and deep ulcers. In 2018, Akpinar et al. found that high NLR and PLR levels were both predictive of endoscopic active UC, but unlike our study, they used the Rachmilewitz endoscopic activity index to describe disease activity (11).

In the present study, we compared the predictive abilities of the NLR, PLR, and SII for clinical outcomes in patients with moderately to severely active UC after VDZ treatment. The SII may be a better predictor of relapse after VDZ treatment than the NLR and PLR. Although all three biomarkers showed significant differences between the sustained response and relapse groups, the SII showed the highest AUC (0.793) with a cut-off value of 602.0 based on ROC analysis. The reason for this difference may be that the SII integrates platelet, neutrophil and lymphocyte counts and can reflect systemic immune inflammation in patients (23). Notably, while the SII demonstrates strong predictive capability, its specificity is relatively low at 57.1%. Therefore, in clinical practice, it should be used in conjunction with the patient’s clinical symptoms, physical signs, and biomarkers such as CRP and ESR to achieve higher predictive accuracy.

Shmidt et al. reported cumulative loss-of-response rates of 18 and 39% at months 6 and 12, respectively, in 195 UC patients receiving VDZ therapy (31). Similarly, the relapse-free survival rates in this study were 89.4 and 60.4% at months 6 and 12 after VDZ treatment, that is, the cumulative relapse rates were 10.6 and 39.6%, respectively. A higher rate of cumulative clinical relapse-free survival was observed in patients with SII <602.0, compared to those with SII ≥602.0. Up to the endpoint of follow-up, 14 of the 17 relapsed patients underwent treatment escalation and had higher SII values than patients who did not undergo treatment escalation. Furthermore, the multivariate Cox regression suggested that SII ≥602.0 was a risk factor for relapse after VDZ treatment. These results suggest that patients with high SII levels at baseline are at risk for VDZ treatment failure, and clinicians should promptly adjust their treatment strategies according to SII levels.

TNF-*α* inhibitors represent a safe and effective class of novel therapeutic agents for managing chronic inflammatory immune-mediated diseases (38). Albayrak et al. demonstrated that NLR, PLR, and SII could serve as reliable biomarkers for monitoring TNF-*α* inhibitor therapy in psoriasis (39). However, the scarcity of studies investigating the association between TNF-*α* inhibitors and SII in patients with UC significantly limits our ability to evaluate the comparative efficacy of VDZ and TNF-*α* inhibitors in modulating SII.

This study had several limitations. First, some patients did not undergo colonoscopy after clinical remission from VDZ treatment, therefore, this study lacked an analysis of the NLR, PLR, and SII correlating with endoscopic remission. Second, fecal calprotectin and VDZ trough concentration levels were not evaluated due to technical reasons, which were found to be correlated with the prognosis of UC patients (40, 41). Third, neutrophil counts are susceptible to corticosteroids, but our results found no significant differences in baseline NLR, PLR, and SII values between steroid-using and non-using subgroups. Finally, this is a single-center retrospective study, so the value of the SII in UC patients treated with VDZ should be confirmed in further prospective studies.

## Conclusion

To summarize, the SII performed better than the NLR and PLR in predicting clinical remission and relapse in patients with moderate-to-severe UC on VDZ therapy and was significantly associated with MES and laboratory parameters. Furthermore, owing to the safety and accessibility of SII, it may be more suitable for application in VDZ follow-up for timely adjustment of the treatment regimen for UC patients.

## Data Availability

The raw data supporting the conclusions of this article will be made available by the authors, without undue reservation.

## Supplementary material

SUPPLEMENTARY FIGURE 1 The comparison of NLR **(A)**, PLR **(B)**, and SII **(C)** between VDZ alone/with 5-ASA (*n* = 47) and VDZ with corticosteroid (*n* = 27) groups. NLR, neutrophil-to-lymphocyte ratio; PLR, platelet-to-lymphocyte ratio; SII, systemic immune-inflammation index; VDZ, Vedolizumab; 5-ASA, 5-aminosalicylates.
