# Supplementary figures and images for: The systemic immune-inflammation index is superior to predicting clinical remission and relapse for ulcerative colitis patients treated with vedolizumab
Source: Front Med (Lausanne). 2025 Mar 13;12:1524307. doi: 10.3389/fmed.2025.1524307 (PMC11965917; doi:10.3389/fmed.2025.1524307)

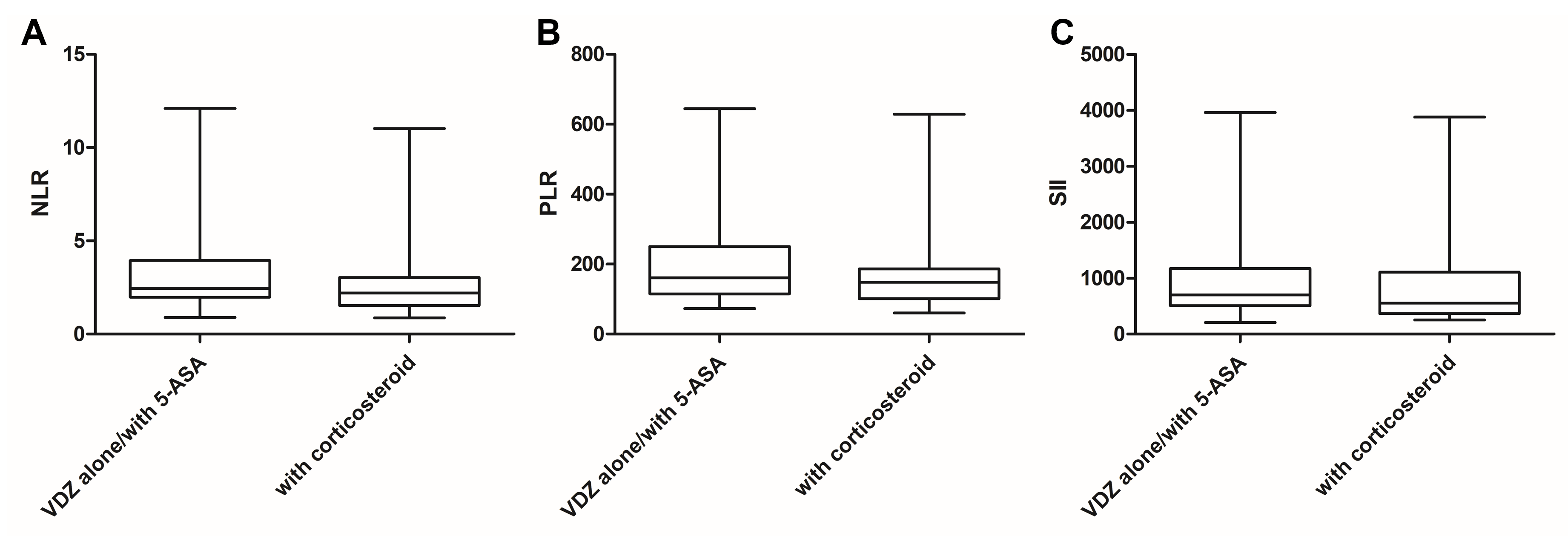

Supplement: SUPPLEMENTARY FIGURE 1 — The comparison of NLR (A), PLR (B), and SII (C) between VDZ alone/with 5-ASA (n = 47) and VDZ with corticosteroid (n = 27) groups. NLR, neutrophil-to-lymphocyte ratio; PLR, platelet-to-lymphocyte ratio; SII, systemic immune-inflammation index; VDZ, Vedolizumab; 5-ASA, 5-aminosalicylates. [file Image_1.TIF]
